# The Potential Therapeutic Role of Celastrol in Patients With Heart Failure With Preserved Ejection Fraction

**DOI:** 10.3389/fcvm.2021.725602

**Published:** 2021-08-18

**Authors:** Maryam Ajmal, Aisha Ajmal, Lei Huang, Lingfang Zeng

**Affiliations:** ^1^GKT School of Medical Education, Faculty of Life Science and Medicine, King's College London, London, United Kingdom; ^2^St. George's Hospital Medical School, University of London, London, United Kingdom; ^3^Department of Heart Center, Tianjin Third Central Hospital, Tianjin, China; ^4^Tianjin Key Laboratory of Extracorporeal Life Support for Critical Diseases, Tianjin Third Central Hospital, Tianjin, China; ^5^School of Cardiovascular Medicine and Sciences, King's College London British Heart Foundation Centre of Excellence, Faculty of Life Science and Medicine, King's College London, London, United Kingdom

**Keywords:** cardiovascular disease, heart failure, diastolic dysfunction, heart failure with preserved ejection fraction, systems disease, celastrol, traditional Chinese medicine, oxidative stress

## Abstract

Analysis of left ventricular systolic dysfunction remained at the centre of heart failure research for many years (also known as heart failure with reduced ejection fraction, HFrEF). Although more than 50% of all heart failure patients experience a form of heart failure characterised by preserved ejection fraction (HFpEF), the pathophysiological mechanisms leading to this form of heart failure remain not well-understood. Several evidence-based treatments for HFrEF are in routine use, but there are limited evidence-based therapies for HFpEF. The effects of these remain controversial, with current treatment options being limited to managing the associated symptoms and conditions. Accumulating evidence demonstrates that pro-inflammatory and oxidative stress pathways play key roles in the development and progression of HFpEF, such as the Unfolded Protein Response (UPR) and inducible nitric oxide synthase. Celastrol, derived from medicinal plants, is a bioactive compound with strong anti-inflammatory properties, which could deem it as fruitful in overcoming the effects of such dysregulated UPR. This literature review therefore focuses on Celastrol's anti-inflammatory and antioxidant activities, alongside its other potential therapeutic activities, and its ability to impede the pathways that are thought to be involved in the development of HFpEF, such as the JAK2/STAT pathway, to elucidate the potential therapeutic role of this bioactive compound, in the treatment of HFpEF.

## Introduction

Heart failure (HF) is often referred to as a complex clinical syndrome and it manifests with a reduction in the ability of the heart to maintain adequate pumping and/or filling with blood (1). From a physiological perspective, HF can be described as the insufficiency of the cardiac output to fulfil patients' metabolic demands (1). Such HF patients may eventually progress to experience distressing symptoms such as dyspnoea and fatigue and can describe themselves as having a decreased quality of life. HF has been described as a global pandemic, with approximately 26 million people being affected globally (2). It remains as one of the leading causes of morbidity and mortality worldwide, with an incidence of 5–10 per 1,000 persons per year, totalling up to 1–2% of the adult population in western countries (3).

Until the 1980s, HF was thought to be a problem of impaired left ventricular systolic function. Since then, it has been found that a considerable number of patients with symptoms of HF have a normal ejection fraction. In the 1991 therefore, the New England Journal of Medicine first defined these patients as experiencing a form of HF known as diastolic heart failure (4). Recently, with the in-depth understanding of the physiological mechanisms underpinning the development of HF, diastolic heart failure is considered to not only be a pathophysiological abnormality that is characterised by left ventricular diastolic dysfunction as the main manifestation, but it also encompasses a variety of pathophysiology and multi-organ dysfunction (5). The European Heart Failure Guidelines therefore pioneered the concept of heart failure with preserved ejection fraction (HFpEF), which is also referred to as “diastolic dysfunction,” and developed updated diagnostic criteria (6). In terms of classification of HF therefore, HF can be thought of as having three subtypes: systolic heart failure (also known as heart failure with reduced ejection fraction), characterised by impaired ventricular contractions; HFpEF, characterised by preserved ejection fraction and impaired ventricular relaxation, and the subsequently impaired ventricular filling; and a newer category of HF, known as mid-range ejection fraction (HFmrEF), characterised by LVEF of 40–49%, elevated natriuretic peptides (BNP ≥ 35 pg/ mL or NT-proBNP ≥ 125 pg/mL), and the presence of a structural heart disease such as, but not limited to, left ventricular hypertrophy (7).

As for HFpEF, it is regarded as a systemic syndrome affected by risk factors and comorbidities; the hallmarks of which are adverse cardiac remodelling and diastolic dysfunction (8). Although the mortality rate from HFpEF is nearly as high as that from heart failure with reduced ejection fraction (HFrEF), the underlying mechanisms are not fully elucidated and strategies for treating this global problem remain limited. Existing treatment options mainly aim to manage the associated symptoms/conditions, such as controlling blood pressure, improving myocardial oxygen supply/demand balance, etc. Several randomised controlled trials (RCTs) have shown that the primary treatments for HFrEF, e.g., renin-angiotensin-aldosterone system (RAAS) antagonist, do not significantly reduce the risk of all-cause and cardiovascular deaths in patients with HFpEF (9). Thus, there exists a need to delve deeper into the pathophysiological processes underpinning the structural/functional changes that are seen in HFpEF, to possibly elucidate a treatment mechanism.

As for the potential mechanisms that underpin the development of HFpEF, myocardial inflammation, oxidative stress, coronary endothelial dysfunction which lead to hypertrophy and stiffness of cardiomyocytes, and enhanced myocardial fibrosis have been proposed recently as impacting factors (10). Investigating pharmacological molecules that inhibit these target pathways would therefore be very beneficial in the treatment of HFpEF. Herein, we therefore begin by presenting an update of the recent advances in the pharmacological agents that are employed‘ in the treatment of HFpEF.

## Pharmacological Advancements for HFpEF

A systemic review and meta analysis published recently showed that the drugs commonly used to treat HFrEF, such as angiotensin-converting enzyme inhibitors, aldosterone receptor blockers and mineralocorticoid receptor antagonists have had no significant effect on reducing all-cause and cardiovascular mortality compared with placebos (11). The emerging perception is that the fundamental pathological changes leading to HFpEF are coronary microvascular endothelial inflammation, epicardial adipose tissue accumulation, myocardial fibrosis and vascular stiffness. Thus, potentially promising treatment options are thought to include anti-inflammatory-inducing mechanisms and by restoring the activity of the associated pathways.

### Anti-inflammation and Restoration of “NO-cGMP-PKG” Axis

Interleukin 1 (IL-1) affects the systolic and diastolic function of the heart, and its related diseases (e.g., rheumatoid arthritis) are often accompanied by impaired diastolic function of the ventricle. Anakinra, a recombinant receptor antagonist of human IL-1, is approved for treating systemic inflammatory diseases. It has been shown to significantly improve the efficiency of ventilation in patients with HFpEF and to reduce serum levels of C-reactive protein and NT-proBNP, thus improving exercise time and quality of life (12, 13). Nitrates, a donor of reactive nitric oxide, enhances the NO-cGMP-PKG signalling pathway. Oral administration of nitrite (e.g., beetroot juice) has shown to improve the exercise capacity and compliance of peripheral vasculature of patients with HFpEF (14). Soluble guanylate cyclase stimulators (e.g., vilicigua) and phosphodiesterase-5 inhibitors (e.g., sildenafil), which enhance the biological activity of cyclic guanosine monophosphate (cGMP) and promote the synthesis of nitric oxide (NO), have also shown to improve haemodynamic parameters, diastolic function of the ventricles, and long-term prognosis and quality of life (15). However, the results of these clinical trials are controversial and need to be further verified (15–18).

### Sodium-Dependent Glucose Transporters 2 (SGLT-2) Inhibitors

SGLT-2 inhibitors are a new type of anti-diabetic drugs and work by inhibiting the reabsorption of glucose by the kidneys. It has been discovered that diabetic patients with cardiovascular disease who were treated with SGLT-2 inhibitors, showed a significant reduction in the incidence of major cardiovascular events and all-cause mortality when compared with the placebo group (19). In favour of the aforementioned discovery, a recent phase III RCT named EMPEROR-Preserved showed that Empagliflozin (SGLT-2 inhibitor) significantly reduced the risk of cardiovascular death or hospitalisation in patients with HFpEF compared with placebo, regardless of whether the patient had diabetes.

### Antifibrotic Agents

Myocardial fibrosis is a key pathological mechanism in HFpEF. Pirfenidone is an anti-fibrotic medication licenced for the treatment of idiopathic lung fibrosis. It's clinical effects are translated by its ability to reduce lung function decline, improve progression free survival and reduce all-cause mortality. In pre-clinical models, pirfenidone was shown to attenuate profibrotic pathways and has been associated with the regression of myocardial fibrosis. Currently, an evaluation of the efficacy and safety of pirfenidone in the treatment of HFpEF remains ongoing *via* a Phase II RCT (20).

### An Enhancer of Myocardial Energy Metabolism

Cardiomyocytes in patients with HFpEF are often accompanied by abnormal energy metabolism (21). Therefore, improving myocardial energy metabolism has become a new focus in the treatment of HFpEF. Elamipretide is a mitochondrial function enhancer, which improves the function of respiratory chain, increases the level of ATP production, and reduces the production of oxidative stress products. Clinical studies of such medical agents, such as elamipretide, in the treatment of HFpEF remain currently underway in the hope of providing a profound theoretical basis for understanding their pharmacological properties (22).

### Traditional Chinese Medicine

*Traditional Chinese Medicine* (TCM) has been used to treat inflammatory diseases for thousands of years, involving multi-level and multi-targeted treatment strategies with fewer side effects. Accumulating evidence revealed that some Chinese herbal medicines have had potential therapeutic prospects for reversing the pathophysiological process of HFpEF, owing to their protective actions against cardiac fibrosis, oxidation and inflammation, such as Resveratrol (23), combination of Fuzi and Banxia (24), and a compound Chinese medicine preparation called “Zhigancao Tang,” the latter of which is still undergoing validation (25).

In our review, we focus primarily on a similarly promising TCM: Celastrol. This medicinal herb is a natural friedelane pentacyclic triterpenoid that is isolated from the root pulp of a Chinese medicinal plant, Tripterygium wilfordii (26). It has attracted a widespread of interest for its remarkable anti-inflammatory and anti-cancer effects (27). In cardiovascular and metabolic systems, accumulating evidence demonstrates the bioactive compound's anti-obesity (28), anti-inflammatory and antioxidant actions (29) and the ability to regulate type 2 diabetes (30)—each of which are implicated in the pathophysiological development of HFpEF. Similarly, the bioactive compound is known to target different pathways that are thought to be involved in the pathophysiological development of HFpEF, such as the JAK2/STAT pathway, which is responsible for transforming growth factor-β1 (TGF-β1) and collagen (31). Likewise increased understanding and accumulating evidence of Celastrol's ability to interact with many cellular targets could highlight the promising therapeutic role of Celastrol in the treatment of HFpEF. Hence, in this review, we will give a concise overview of the advancements in the understanding of the pathophysiological mechanisms that lead to HFpEF, and we will provide an insight as to whether such a bioactive compound can potentially be used to in the treatment/prevention of HFpEF.

## The Pathophysiological Development of HFpEF

### Mechanical Stress Disorder

Arterial hypertension is the most common comorbidity in patients with HFpEF. HFpEF is generally considered as a form of hypertensive heart disease–supported by the evidence that pressure overload leads to left ventricular concentric hypertrophy, fibrotic remodelling, and diastolic dysfunction (32). Mechanically, pressure overload activates the immune cascade, such as p38-MAPK, leading to increased T lymphocytes activation, spillovers of cytokines and transformation of macrophages into proinflammatory phenotype of M1, leading to and sustaining increased inflammatory and oxidative stress damage to myocardial tissue and the vascular endothelium (33).

The interaction between left and right ventricles remains as another important pathophysiological mechanism, as several studies have found that obese patients with HFpEF have increased cardiac volume and epicardial fat, which strengthens the restriction of pericardial restrain on ventricles and leads to the deviation of ventricular septum from left to right (34). One recent study has found that even in non-obese patients, exercise-induced pulmonary hypertension also increases the interaction between left and right ventricles (35).

### Inflammatory Disease

Importantly, HFpEF has been widely accepted as an inflammatory disease (36, 37). Previous experiments have revealed that the myocardial inflammatory response plays a pivotal role in the development of HFpEF. In an animal experiment, circulating levels of the pro-inflammatory cytokines TNF-α, IL-6, and IL-1β were markedly increased in HFpEF mice compared to sham mice (38). Furthermore, HFpEF patients showed higher levels of plasma inflammatory cytokines IL-6 and TNF-α than the healthy population (39). Most patients with HFpEF have several pro-inflammatory comorbidities, such as obesity, hypertension, diabetes and chronic obstructive pulmonary disease, where inflammation, oxidative stress and endothelial impairment are often involved in the lead-up to myocardial hypertrophy or increased stiffness.

One such inflammatory disease includes diabetes. Insulin resistance and hyperglycaemia also promote inflammation. These exert their effects through the upregulation of a series of proinflammatory signalling pathways, such as nuclear factor kappa B (NF-kB). As such, Ju et al. demonstrate the upregulation of pro-inflammatory mediators as contributing to β-cell destruction during development of type 1 diabetes mellitus. Their results highlight the implication of such proinflammatory mediators as encouraging the enhanced infiltration of immune cells into pancreatic islets (40). Within the cardiomyocytes then, the excessive release of pro-inflammatory cytokines and inflammasome, results with upregulated ROS production, and downregulated signalling pathways that are involved in anti-inflammation and glucose homeostasis, ultimately leading to cardiac dysfunction (41).

COPD is also a chronic, systemic inflammatory disease, which is closely related to cardiovascular disease, particularly HF. Local, low intensity, inflammatory cytokines in the pulmonary circulation recruit the adhesion of leukocytes to the pulmonary vessels, inducing the iterative production of ROS and cytokines, eventually leading to haemodynamic stress. Inflammatory cytokines that enter the systemic circulation also act directly on the myocardium, promoting adverse cardiovascular remodelling (42). Finally, the systemic pro-inflammatory state induced by HFpEF results in coronary microvascular endothelial inflammation, reduced nitric oxide (NO) bioavailability, decreased cyclic guanosine monophosphate (cGMP) content and decreased protein kinase G (PKG) activity, leading to eventual endothelial dysfunction (36, 43). Impairment of endothelial function also increases the level of TGF-β1, promotes the transformation of fibroblast into myofibroblast, and increases the deposition of collagen in the intercellular space and fibrosis (43, 44).

Recent advances in this field have led to the discovery that the accumulation of epicardial fat plays an essential role in the pathogenesis of HFpEF, promoting inflammation of deep tissues, fibrosis and adverse arrhythmogenesis at a paracrine level (45, 46). This contribution is only present in HFpEF but not HFrEF, and is independent of key anthropometric indicators (e.g., abdominal subcutaneous fat and visceral fat), haemodynamic stress indicators and race (45).

### Oxidative Stress

Oxidative stress could represent joint predisposing processes with inflammation. In physiological conditions, intracellular reactive oxidative species (ROS) in controlled concentrations are vital for facilitating redox signalling that are involved in the maintenance of vascular function and integrity. However, when the production of these ROS overwhelm the cellular antioxidant defending capacity, this contributes to vascular dysfunction and remodelling, through oxidative damage (47). The implication of such ROS-mediated oxidative stress in the pathophysiology underpinning cardiovascular disease is often attributed to induced abnormal cell signalling, inflammation, hypertrophy, proliferation and fibrosis (48–50). In vascular cells, the outcome is often vascular remodelling and endothelial dysfunction, which when coupled with hypertension, atherosclerosis, obesity or diabetes, can often link to HFpEF (48).

#### NADPH Oxidase (NOX)-Derived Oxidative Stress

The cascade of events results with the exacerbation of oxidative stress from ROS production, leading to a decrease in the synthesis of the vascular relaxant, NO, and consequently, increased oxidative stress (51). Several enzymes have been identified as being responsible for ROS production in various tissues, often differing at the molecular level. The most notable ones are those commonly grouped together as the NADPH oxidase (NOX) family. These, often resembling phagocytic NOX (NOX2, also known as gp91^*phox*^), are characterised by their NOX genes–the presence of which leads to the production of transmembrane proteins responsible for transporting electrons across biological membranes (47). This is a crucial step in facilitating superoxide generation from reduced oxygen (52). NOX proteins are associated together due to their conserved structural properties such as the NADPH-binding site at the C-terminus, their four heme-binding histidines and their six transmembrane domains. Although sharing conserved similar features, the different isoforms of NOX are clearly distinguishable by their interacting proteins and specific catalytic subunits (51). NOX enzymes mediate diverse functions in various organisms through redox signalling but generally require association of the catalytic subunit with other proteins. For example, the catalytic subunit NOX2 often depends on at least five subunits including: p47^*phox*^, p67^*phox*^, p40^*phox*^ and p22^*phox*^ (51).

The implication of NOX 2-derived oxidative stress in the pathophysiology of HFpEF can be elaborated on using models of experimental hypertension, atherosclerosis and ischemia-reperfusion injury, which demonstrate the subsequent upregulated levels of vascular NOX2 (47, 51). As such, existing data show the implication of oxidative stress-induced inflammatory influence (usually *via* NOX-generated ROS) (53) in the development of diastolic heart failure, through the influence on the structure, sub-cellular localisation and affinity for DNA of the transcription factors encoding the pro-inflammatory genes (53). The resultant hypophosphorylation of titin impairs left ventricular relaxation, further leading to the development of diastolic heart failure (54). Moreover, such (chronic) inflammatory-mediated pathologies often result with the infiltration of monocytes into cardiac tissues and their differentiation into macrophages. Here, monocytes often secrete transforming growth TGF-β, whereby promoting fibrosis by differentiation of fibroblasts into myofibroblasts (54).

#### Ang-II-Derived Oxidative Stress

Angiotensin (Ang)-II could possibly play a role in maintaining vascular and physiological homeostasis, by inducing vasoconstriction to regulate the blood pressure and by also regulating fluid and electrolyte homeostasis. Often such vasoconstriction-dependent-induced hypertension and vasoconstriction-independent pathogenesis can result with excess ROS production, which adversely results with cardiac remodelling and signalling pathways interruptions. Numerous studies have already elaborated on the association between Ang-II and its role in increasing ROS production in the failing heart, often through left ventricular hypertrophy and adverse cardiac remodelling. Interestingly, one such study demonstrated doubled AT1R (Angiotensin 1 receptor)-dependent hydroxyl production in mice hearts after receiving infusion with Ang-II for 2 weeks, which causes cardiac hypertrophy (55). Moreover, human patients with HF demonstrated increased oxidative stress markers, which correlated with circulating Ang-II levels–the highest of which were expressed in patients homozygous for the *AT1R* A1166C gene polymorphism, and therefore expressed increased AT1R responsiveness to Ang-II (56). ACE-inhibitor therapy, although routinely used in the treatment of hypertension and HF, does not completely block Ang-II production, and some patients can often show elevated Ang-II levels despite ACE-inhibitor therapy. Often, this can be due to the conversion of Ang-I to Ang-II by chymase activity (57).

#### Nitrosative Stress

Oxidative stress-induced cardiovascular signalling failure is heightened in the cardiovascular system by its influence on the formation of the monomeric, uncoupled form of NOS (nitric oxide synthase) from its dimeric, stable form. All three NOS isoforms have shown susceptibility to “uncoupling” under certain conditions, leading to a common end state as superoxide (rather than NO) (58). As for endothelial nitric oxidase synthase (eNOS), a NOS isoform, its implication in the pathophysiology of HFpEF is linked with conditions of ROS-mediated oxidative stress. The oxidation of tetrahydrobiopterin, when subjected to oxidative stress, seems to be the critical step in facilitating this development toward HFpEF. As such, the oxidation of tetrahydrobiopterin results with a decrease in bioavailability of this cofactor, and the subsequent decrease in NO production, as demonstrated by Landmesser et al. (59). Their findings indicate the end result of such a hypertension-induced-cascade as involving NADPH oxidase-induced ROS production, the subsequent oxidation of tetrahydrobiopterin and the uncoupling of eNOS. The uncoupling of eNOS is characterised by the L-arginine oxidation and subsequent electron transfer from NOS flavins and the ferrous-dioxygen complex dissociation, and the superoxide production from the oxygenase domain (60). This process has been duplicated *in vitro* through the absence of the co-factor tetrahydrobiopterin (59). Importantly, mice with deoxycorticosterone acetate (DOCA) salt-induced hypertension also demonstrated evidence for hypertension-induced uncoupling of eNOS.

In the vasculature, the plausible end result of likewise decreased NO is often hypertension, characterised by vasoconstriction and increased susceptibility to mechanical forces, such as oscillatory shear. The presence of likewise mechanical forces is linked to increased oxidative damage, as in hypertension (61). Direct measurements of ROS production from stimulated mononuclear cells showed cells measuring higher levels of superoxide production following stimulation with angiotensin-II when compared to normotensive subjects (62). Such mononuclear cells were those isolated from hypertensive patients (62). These findings elaborate on the role of ROS in setting up the vicious cycle between ROS-induced-hypertension and the resultant ROS production, from hypertension induced mechanical stress.

As for inducible nitric oxidase synthase (iNOS), a NOS isoform, it is one of the key enzymes that are implicated in the generation of NO from the amino acid, L-arginine (63). Usually iNOS-derived NO is of significance in regulating physiological and pathophysiological conditions, such as, inflammation and infection. Within the cardiac setting more specifically, the study of Schiattarella et al., is of pioneering significance, as they highlight the role of iNOS in underpinning adverse cardiac remodelling. They report mice fed with a high fat diet (HFD) and L-NAME, when given an inhibitor of iNOS, showed increased ventricular relaxation (64). Mechanistically, the activity of iNOS results with the S-nitrosylation of the Inositol Requiring Protein 1-alpha (IRE1-alpha), and the consequent downregulation of X-Box Binding Protein 1 spliced isoform (XBP1s) (64). XBP1s regulates the degradation of abnormally folded proteins, a process known as endoplasmic-reticulum-associated protein degradation (ERAD), as it translocates to the nucleus and influences the upregulation of a subset of UPR-related genes linked to protein folding, quality control and ER/Golgi biogenesis (65). Schiattarella et al. show this phenomenon as being unique to HFpEF and not HFrEF, as endomyocardial biopsies of control and failing human hearts showed XBP1s transcript as being reduced in human HFpEF hearts, whereas the unspliced form of XBP1 remained unchanged in HFrEF hearts.

The detrimental effects of downregulated transcriptional factors, such as XBP1s, modulating the ER stress response can be elaborated by the severe ER stress seen in the hypothalami of neuronal XBP1-deficient mice upon HFD feeding (66, 67). The consequential increased ER stress, severe hyperleptinemia, leptin resistance, and obesity seen in HFD mice (28) highlight the role of such impaired UPR response in the development of HFpEF.

Comorbidities such as diabetes and obesity can also influence the implication of the UPR and the subsequent nitrosative stress in leading to the development of HFpEF. As for diabetes, dysregulated UPR signalling of islet β-cell has been suggested to result with the hyperactivation of IRE1α in β-cells, whereby promoting β-cell early autonomous apoptosis, and thus, being implicated in the development of postnatal diabetes in this manner (68). Studies have therefore focused on pharmacologically attenuating IRE1α activity to halt the progression of diabetes. Imatinib has been on such pharamacological agent, which has shown to be effective in blunting the hyperactivity of IRE1α, whereby reducing pancreatic β-cell apoptosis, and reversing type 1 diabetes (T1D) in diabetic patients (68). Diabetes is it is said to be associated with protein nitration due to activation of iNOS in the endothelium (69). From a pathophysiological perspective, nitrosative stress, as induced by peroxynitrite (amongst other nitrosative stress-inducing species) can influence the activation of NF-κB, which promotes the transcription of inflammatory cytokines the likes of IL-1β and TNF-α (70). Emerging studies therefore assert that the inhibition of iNOS expression may serve as a protective factor for diabetic patients from the damage caused by nitration of proteins (70).

Obesity, also another comorbidity, acts as a major influencing factor for the development of debilitating conditions such as type 2 diabetes and hypertension (71), which can be incorporated in the pathophysiology of HFpEF (72). Like diabetes, obesity has also been demonstrated to be associated with a failure of the UPR or the ERAD pathway, whereby resulting with low-grade systemic inflammation, which may contribute to the onset and development of HFpEF. As such, 2 weeks of XBP1s administration into mice on a HFD and L-NAME demonstrated ameliorated diastolic dysfunction and decreased expression of genes that are implicated in HF (64). A therapeutic proposal would therefore be to focus on strategies that minimise the adverse effects of nitrosative stress, such as increasing the expression of XBP1s, to overcome the adverse effects in underpinning the pathophysiological changes, which lead to HFpEF.

### Other Potential Mechanisms

Recently, accumulating evidence has demonstrated that HFpEF syndrome has been echoed by several other mechanisms, including the heart itself (e.g., chronotropic incompetence) (73, 74), or the factors outside the heart (e.g., abnormal ventricular-arterial coupling) (75, 76), exercise-induced pulmonary hypertension (77) and systemic volume overload (78, 79). These complex pathophysiological mechanisms suggest that HFpEF might be a highly heterogeneous disease entity, which may be a disease of the heart itself, or it may be the manifestation of systemic diseases in the heart.

## Celastrol's Role in Potentially Providing Therapeutic Relief in HFpEF

### Overcoming iNOS-Induced Nitrosative Stress

Mechanistically, the activity of iNOS results with the S-nitrosylation of the Inositol Requiring Protein 1-alpha (IRE1-alpha), and the consequent downregulation of X-Box Binding Protein 1 (XBP1) (64), and increase in PERK phosphorylation. The therapeutic role of Celastrol in overcoming the iNOS-mediated HFpEF can therefore be eluded to when considering the (mild) activation seen in the levels of PERK in mice subjected to HFD and L-NAME (64). The increase in PERK activity is associated with increased ER stress, as also elaborated on by Nakato et al., who show that NO can S-nitrosylate PERK by activating its kinase activity (80). Celastrol's ability to significantly decrease PERK phosphorylation, as seen in the study of leptin and obesity (28), therefore provides a direct link between Celastrol, decreased ER stress and increased leptin sensitisation. Since Celastrol has shown to decrease PERK phosphorylation (28), this sets the scene for Celastrol to potentially overcome the nitrosative stress-induced-dysfunctional ER stress signalling, and its subsequent role in regulating the pathogenesis underpinning HFpEF.

Emerging studies have also demonstrated Celastrol as capable of inducing a terminal UPR in the cell lines of oral squamous cell carcinoma (OSCC), through increased levels of UPR-related mRNA transcripts and XBP1 splicing (81). Although the study looked at OSCC primarily, the results suggest that this phenomenon is not unique to OSCC, as the IC50 values for the neuroblastoma cell lines SH-SY5Y and SH-BE were similar to OSCC cells with intact TGFβR/SMAD4 (81). Should the results be replicable in the hypothalami of mice/human cells also, the induction of the UPR-related mRNA transcripts in the hypothalami could render Celastrol potent in reducing leptin resistance and hyperleptinaemia.

### Overcoming eNOS-Induced Nitrosative Stress

When considering the oxidative-stress-induced-uncoupling of eNOS, Silberman et al. (82) demonstrate short-term treatment with tetrahydrobiopterin in isolated cardiomyocytes from hypertensive mice as improving impaired relaxation. Similarly, their results show that feeding hypertensive mice tetrahydrobiopterin (5 mg/d) improved cardiac tetrahydrobiopterin stores and diastolic dysfunction.

**Figure 1:**
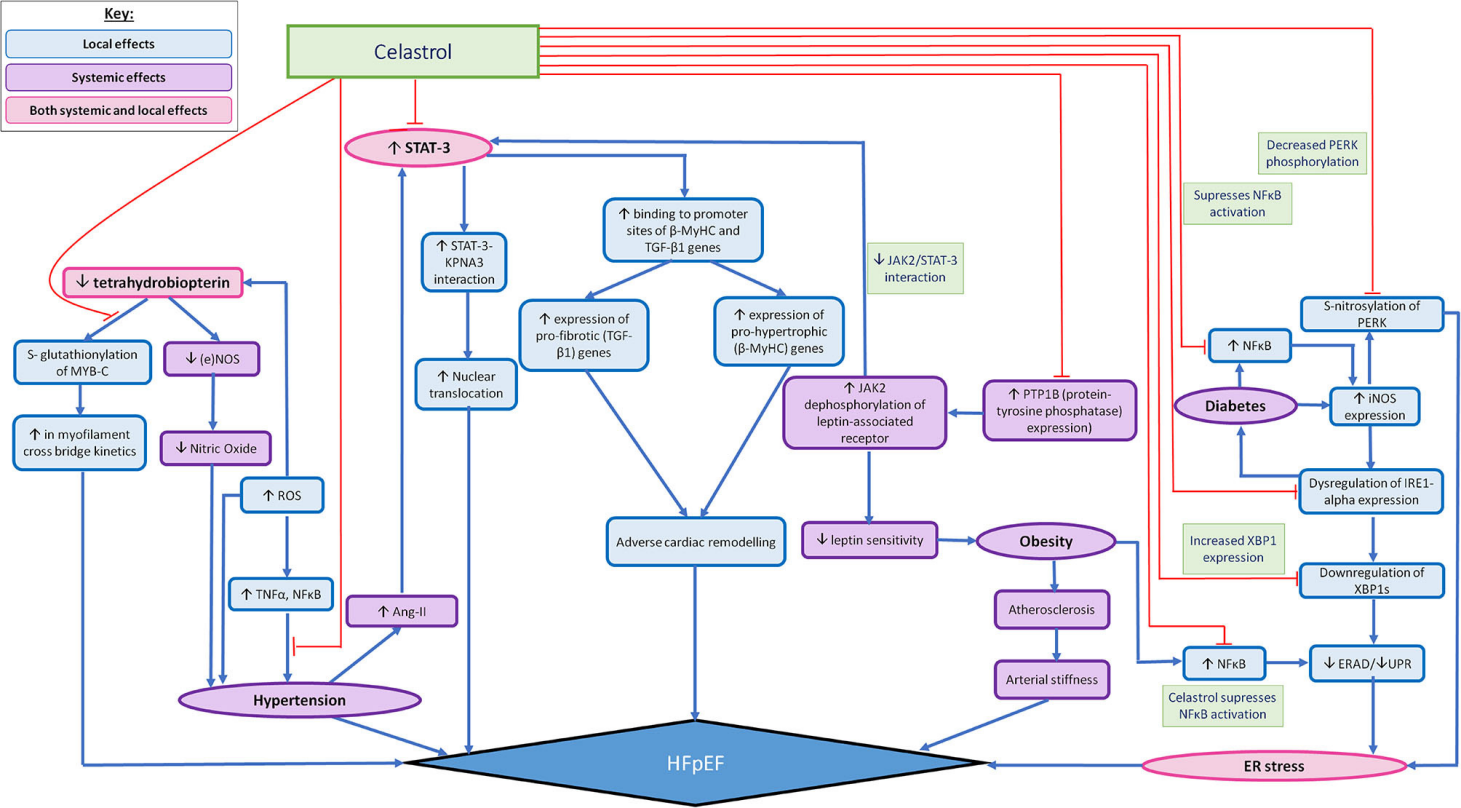
Summarising the possible widespread effects of Celastrol in mediating localised (in relation to the heart) and systemic pathophysiological mechanisms that are thought to lead to HFpEF. The pathophysiological processes that lead to the development of HFpEF are demonstrated by the pathways illustrated with blue arrows. The red arrows outline the pathways which can be altered by the action of Celastrol, so as to impede the pathophysiological development of HFpEF. The local effects of Celastrol are demonstrated by the blue (accent 5) boxes, the systemic effects are demonstrated by the purple boxes and the combined local and systemic effects are demonstrated by the pink boxes.

The usefulness of such findings in the clinical setting can be amplified when considering them as potential targets in the prevention of HFpEF, as eNOS-based superoxide generation has often been implicated in experimental and clinical vascular disease states (83). Likewise superoxide induced diseased states constituting as risk factors for HFpEF include diabetes (84), cigarette smoking (85), hypertension (59) and atherosclerosis (86) and Celastrol has proven effective in modifying such pathways. The mechanisms of action of Celastrol have been summarised in (Figure 1).

### Overcoming the Oxidative Stress-Induced Cardiac Pathologies

Notable impacts of the adverse effects of oxidative stress in cardiac signalling pathway-interruption include the activation of tumour necrosis factor-α (TNFα), the activation of NF-kB (50, 87, 88) and the role in upregulating endothelial chemokines and chemoattractant molecules. The result is the triggering of an inflammatory response in models of HFpEF and MI (89, 90), leading to an oxidative stress-induced inflammatory influence on the structure, sub-cellular localisation and affinity for DNA of the transcription factors encoding the pro-inflammatory genes. Celastrol's ability to reduce the production of pro-inflammatory cytokines such as TNF-α and IL-6 (31) allude it as having great therapeutic benefit when questioning its ability to reduce the influence of oxidative stress in causing inflammation-induced cardiac pathologies.

Interestingly also, models of calcific aortic valve disease-induced rats highlight Celastrol as a direct preferable NOX2 inhibitor through its ability to inhibit the NOX2-mediated GSK3B/b-catenin pathway (91). As described previously, models of experimental hypertension, atherosclerosis and ischemia-reperfusion injury showed upregulated levels of vascular NOX2 (derived from resident macrophages or vascular cells) (47, 51). The end result of such upregulated NOX2 levels is often characterised with increased section of TGF-β by monocytes, and the subsequent promotion of fibrosis, leading to a diseased heart state (54).

Interestingly however, despite not being described in the vasculature, the absence of NOX-2-dependent oxidative burst has led to the abnormally increased inflammatory states in models of arthritis (92). Consequently, if Celastrol is to be used to target oxidative stress-induced cardiac diseased states, a more profound and clear understanding of the role of NOX-derived ROS species in the pathogenesis of inflammation is required.

### Overcoming Ang-II-Induced Cardiac Pathologies

Celastrol's therapeutic potency is enhanced when considering its ability to inhibit signal transducers and activators of transcription-3 (STAT-3) that are activated by Ang-II, downstream of ATR1 (31). It does this by preventing the induction of STAT-3 target genes such as those that are responsible for regulating TGF-β and collagen, whereby potentially mediating the pathways leading to such structural and functional changes that progress to a diseased myocardium and the subsequent (diastolic) dysfunction.

### Enhancing the Function of Tetrahydrobiopterin in Mediating Redox Homeostasis

The presence of Ang-II-induced ROS production can heighten the extent of cardiovascular pathologies when considering the oxidative stress-induced impaired endothelium-dependent vasodilatation. As such, Higashi et al. (93) highlight patients with renovascular hypertension (with elevated plasma Ang-II levels) as demonstrating increased oxidative stress together with impaired endothelium-dependent vasodilatation (*via* decreased NOS production). After renal-artery angioplasty, serum and urine levels of several markers of oxidative stress were significantly reduced, accompanied by an significant increase in peripheral blood flow via an enhanced response to acetylcholine, an endothelium-dependent vasodilator (93).

When considering the ROS-mediated oxidation of tetrahydrobiopterin, Celastrol may be of therapeutic use in modulating the efficacy of tetrahydrobiopterin's functioning as a cofactor in the synthesis of eNOS. Antoniades et al. demonstrate hypertension-induced HFpEF as being accompanied by cardiac tetrahydrobiopterin depletion, NOS uncoupling, and the Serine-glutathionylation of MyBP-C. The mechanism by which tetrahydrobiopterin ameliorates HFpEF is attributed to the prevention of Serine-glutathionylation of MyBP-C (94) and the consequent reversal to the changes of myofilament properties that occur during HFpEF, such as depression in myofilament cross-bridge kinetics (95). Celastrol's role as a natural anti-tumour agent accompanies the inhibition of a MyBP-C (96). MyBP-C has directly been implicated in the cardiac vasculature, as Lovelock et al. demonstrate a significant increase in the glutathionylation of MyBP-C in animals with HFpEF. The MyBP-C is thought to directly interact with actin in the thin filaments, causing increased cross-bridge kinetics (97, 98). Since Celastrol is also known to target the MyBP-C (96), perhaps further research into the mechanism by which this targeting occurs could reveal Celastrol as a therapeutic target in lowering the (oxidised) tetrahydrobiopterin-mediated cardiac dysfunction, when subjected to hypertension-induced oxidative stress. Even more so, to enhance the validity of the study, the effects of Celastrol could be studied in the vasculature directly, using cardiac-MyBP-C.

## Critical Discussion

The direct link demonstrated between glutathionylation of cMyBP-C and diastolic dysfunction in hypertensive mice models using DOCA-salt (94), could certainly be useful for prompting the investigation of Celastrol as a therapeutic agent in lowering the (oxidised) tetrahydrobiopterin-mediated cardiac dysfunction. However, it is important to note that such study designs are not free from the indirect effects of other post-translational modifications which may have occurred. Consequently, it is important to control for these in future studies when investigating the effects of Celastrol using cMyBP-C.

Furthermore, although the decreased production of eNOS has been associated with HFpEF, Schiattarella et al. demonstrate the absence of changes in eNOS transcripts in the myocardium of mice subjected to HFD+L-NAME, but an increased iNOS transcript and protein levels (64). Although this indicates the limited implication of eNOS in the development of HFpEF, numerous studies highlight the association between the eNOS uncoupling-induced hypertension, and cardiomyocyte structural and functional changes. Moreover, schiattarella et al. highlight L-NAME as a more potent inhibitor of eNOS when compared to iNOS and so measuring eNOS transcripts in this manner may not entail a fair comparison. Consequently, future comparisons between eNOS and iNOS should employ drivers of endothelial dysfunction-based hypertension that have similar inhibiting effects on both iNOS and eNOS to encompass the involvement of both agents. Furthermore, it is important to consider the context of investigation and whilst Schiattarella et al. mainly focussed on the eNOS transcripts and protein, eNOS transcripts may not encompass the role of eNOS-induced hypertension. Alternative methods may involve directly focussing on hypertension-induced ROS production and the subsequent structural and functional changes leading to a diseased myocardium.

When considering Celastrol's therapeutic role in replacing the administration of tetrahydrobiopterin when treating impaired endothelial relaxation, of importance is the study highlighting Celastrol's role as an anti-cancer agent in patients with colorectal cancer (CRC) (99). Whilst the proposed mechanism of NOS' suppression is useful in suppressing the angiogenesis pathway (99), the results may suggest limited ability of Celastrol in upregulating the activity of eNOS. However, such findings utilise two CRC cell lines, HT29, and HCT116 and may not reflect Celastrol's interaction in cardiac cells. As such, stimulation of epinephrine to cardiac beta-2-adrenoreceptors in the heart, may contribute to an additional increase in heart rate and/or contractility (100, 101) whereas catecholamines' stimulation of beta-2-adrenoreceptors in the lungs trigger a vasodilatory action (102). Hence, more research is required into the pathway by which Celastrol interacts with cardiac cells and its role in regulating eNOS found within the cardiac vasculature to determine the therapeutic usage of Celastrol in mediating ROS-induced eNOS uncoupling.

Moreover, the study described above (99) employs MST assays to measure the amount of NO produced, in order to determine the NOS production. However, MST assays are indirect, indicative of NO oxidative products as they only measure nitrite. Furthermore, nitrates can also interfere with nitrite measure, as nitrates are reduced to nitrites by facultative bacteria in the tongue and reductase enzymes of bacteria in the GI tract (103, 104). Since the study did not control the diet of the participants, the true measure of Celastrol on NOS activity may not be accurately represented. Suggestions for improvement include controlling the diets of participants prior to extracting cells and using more specific, direct NO measurement techniques such as, ESR after *in vivo* or *ex vivo* spin trapping.

When considering Celastrol's therapeutic usage in regulating the Ang-II-induced pathways leading to adverse cardiac remodelling, this may be limited when considering the role of Ang-II in causing HFpEF. Whilst AT1R activation can promote cardiac fibrosis, AT2R may counter-regulate AT1R effects by exhibiting antiproliferative actions in vascular smooth muscle cells. Emerging studies investigating the stimulation of AT2R on fibroblasts extracted from cardiomyopathic hamsters highlight the antifibrotic and antiproliferative effects of AT2R (105). This concept is supported by the transfection of an AT2 expressing vector into the balloon-injured rat carotid artery and the subsequent overexpression of AT2 in attenuating neointima formation (106). This suggests the possibility that AT2 may mediate anti-proliferative effects under physiological or pathophysiological conditions. However, this hypothesis still remains a disputed one as several studies favour not considering AT2 signalling as being essential for adverse cardiac remodelling and HF development. As such, mice with genetic manipulations of the AT1R and AT2R subtypes showed no obvious development of abnormal cardiac remodelling (107). Consequently, if Celastrol is to be used to mediate the Ang-II induced development of HFpEF, the controversy in the activation of AT2R and its impact on the pathways leading to HFpEF needs to be resolved more first (107).

Finally, it's not surprising that many kinases/proteins and signalling pathways like STAT-3 have multi, or opposed actions depending on the disease settings, context, duration of drug treatment and progression of the disease (acute or chronic stress). When considering STAT-3 in the context of leptin satiety, STAT-3 is known to be beneficial in reducing ER stress. However, within the myocardium, preventing the induction of STAT-3 target genes such as those that are responsible for transforming growth factor-β1 and collagen, is useful in preventing the structural and functional changes leading to HFpEF. Furthermore, although STAT-3 has been shown to be effective in mediating Ang-II–induced heart injuries (108, 109) and the inhibition of STAT-3 reverses these injuries (110), gene knockout mice demonstrated STAT-3 deficiency as aggravating to Ang-II–induced heart injuries (111). The differing findings may be context-dependent and could imply the important role of STAT-3 in HFpEF development (112). Consequently, when considering the potency of Celastrol as targeting STAT-3 mediated cardiac dysfunction, it's important to note the context of the disease setting and the role of STAT-3 in facilitating such progression.

## Perspectives

Given the localised (in relation to the heart) and systemic effects of HFpEF, Celastrol may be particularly useful when considering its ability to target multiple pathways that are implicated in the pathophysiology leading to the development of HFpEF, as illustrated in Figure 1. Celastrol's widespread role in mediating the adverse pathophysiological processes leading to HFpEF—both the localised and systemic effects, highlight its somewhat promising therapeutic benefit. As such, Figure 1 shows the multitudinous pathways through which Celastrol may interrupt/redirect cellular and molecular events to limit disease progression and facilitate recovery, whereby shedding light on the possible usage of Celastrol in the treatment/prevention of HFpEF.

Figure 1 also takes into account the role of comorbidities, such as diabetes and obesity, when acting as influencing factors in the development of HFpEF, through their various influences on the development of HFpEF, such as their ability to promote the expression of proinflammatory mediators such as NF-κB. Since existing studies have shown Celastrol as being capable of significantly restoring cytokine-induced cell death and inhibiting cytokine-induced NO production, Celastrol's therapeutic role in reducing the production of pro-inflammatory mediators, such as iNOS (40) have been eluded to already. As such, Celastrol has been demonstrated as being capable also, of ameliorating cytokine toxicity and pro-inflammatory immune responses by its ability to suppress NF-κB activation in rat pancreatic β-cell line (113). Similarly, Celastrol's ability to inhibit NF-κB activity in a type 2 diabetic animal model serves yet another example of Celatrol's therapeutic role in transiently lowering blood glucose levels and inhibiting insulin resistance and diabetic nephropathy (40). We therefore contend further investigation into Celastrol's therapeutic activities could prove it effective in mediating the pathophysiological development of HFpEF, as well as capable of reducing the progression of HFpEF that is otherwise seen as a result of comorbidities such as diabetes and obesity.

To understand more comprehensively, the therapeutic role of Celastrol in the aforementioned ways, more understanding is required however, regarding the discrepancies that pertain the pathophysiological mechanisms that are involved in the pathophysiological development of HFpEF, such as the exact mechanism by which Celastrol acts on cardiac-MyBP-C to determine whether Celastrol could be used to aid/replace entirely, the action of tetrahydrobiopterin in reducing impaired endothelial relaxation. Furthermore, to obtain a true representation of Celastrol's therapeutic benefits, future research using human models is required, as existing studies have mainly utilised animal models, with varying structural and functional cellular and molecular composition, when compared to humans. Consequently, the interaction of Celastrol with human tissues could render different findings. Additionally, in terms of the influence of comorbidities of HFpEF in reducing its efficacy, although Celastrol can be effective at restoring the RNA endonuclease activity of IRE1α and upregulation of XBP1s expression, while correcting the imbalance of UPR, it may lead to the possibility of excessive apoptosis of β cells. Therefore, this could warrant further research on the efficacy and adverse effects of Celastrol in patients with HFpEF and diabetes/likewise comorbidities. Furthermore, Celastrol's clinical application and evaluation of its toxicity also require investigation, as doses reaching 3–4 mg/kg may produce adverse effects (114). We are however delighted to see that advancements in the pharmacology and bioengineering have gradually broken these limitations (115, 116). The development of new drug delivery methods, such as nano-encapsulation, makes the release of Celastrol more targeted, more controllable and durable (116). Future research may therefore focus on experimenting possible modified forms of Celastrol to limit toxicity or on offering combinatorial therapies—the results of which could provide therapeutic breakthroughs in the prevention and treatment of HFpEF.

## Author Contributions

MA, AA, LH, and LZ were involved in the critical evaluation and intellectual contribution to the manuscript. MA, AA, and LH contributed to the literature review and data analysis. MA and AA contributed to devising the diagram. All authors contributed to the article and approved the submitted version.

## Conflict of Interest

The authors declare that the research was conducted in the absence of any commercial or financial relationships that could be construed as a potential conflict of interest.

## Publisher's Note

All claims expressed in this article are solely those of the authors and do not necessarily represent those of their affiliated organizations, or those of the publisher, the editors and the reviewers. Any product that may be evaluated in this article, or claim that may be made by its manufacturer, is not guaranteed or endorsed by the publisher.
